# Elevated creatinine kinase levels amongst Dutch adolescents with acute alcohol intoxication

**DOI:** 10.1007/s00431-023-04820-9

**Published:** 2023-01-20

**Authors:** Louise Pigeaud, Loes de Veld, Nico van der Lely

**Affiliations:** 1 Department of Pediatrics, Reinier de Graaf Gasthuis, P.O. Box F5011, Delft, 2600 GA The Netherlands; 2 Erasmus School of Health Policy & Management, Erasmus University, Rotterdam, 3062 PA The Netherlands; 3 Faculty of Medicine and Health Science, University Antwerp, Prinsstraat 1, Antwerp, 2000 Belgium

**Keywords:** Acute alcohol intoxication, Alcohol-induced rhabdomyolysis, Rhabdomyolysis, Adolescents, Alcohol

## Abstract

This study aims to explore the prevalence of creatinine kinase elevation amongst a sample of Dutch adolescents admitted for acute alcohol intoxication. The data on all admitted adolescents < 18 years old with acute alcohol intoxication between 2008 and 2021 were collected from a Dutch major district general hospital, Reinier de Graaf Gasthuis, in Delft. Overall, 495 adolescents who were treated for symptoms of acute alcohol intoxication during this period were included in the study. When evaluating the blood samples of the included patients, elevated creatinine kinase levels were found in 60% of the cases, with a mean of 254 U/I (normal value ≤ 145 U/I). A confirmed diagnosis of rhabdomyolysis (increase in CK > fivefold the upper limit of normal) was present in 4.4% of cases. Moreover, using a linear regression this study found that a higher blood alcohol concentration was associated with higher creatinine kinase levels, when adjusted for positive drug screenings amongst the adolescents with acute alcohol intoxication (p = 0.027; β = 66.88; 95% CI 7.68 − 126.08).

*Conclusions*: This is the first study focusing on how acute alcohol intoxication affects adolescents’ muscle tissue. The results could potentially help to prevent alcohol use within the sports world. It could also aid understanding of how acute alcohol intoxication influences the breakdown of adolescents’ muscle tissue.

**Table:** 

**What is Known:** *• Alcohol, alongside pharmaceutical agents and illicit drugs, is a significant cause of rhabdomyolysis (increase in creatinine kinase > fivefold the upper limit of normal). * *• Creatinine kinase elevation in alcohol intoxicated patients may be as a result of direct “muscular” toxicity” (myotoxicity) or from prolonged immobilization and ischemic compression induced by coma.*	
**What is New:** *• Our retrospective cohort study is a pioneer in addressing the effect of acute alcohol intoxication amongst adolescents (< 18 years) upon muscle tissue (creatinine kinase level) within a large population. When evaluating the blood samples of the included population, elevated creatinine kinase levels were found in 60% of the cases, with a mean of 254 U/I (normal value ≤ 145 U/I).* *• There is an association between alcohol intoxication and elevated creatinine kinase levels amongst adolescents. Future research is needed to further understand the pathophysiology and causality of this interaction.*

**What is Known:**

*• Alcohol, alongside pharmaceutical agents and illicit drugs, is a significant cause of rhabdomyolysis (increase in creatinine kinase > fivefold the upper limit of normal).
*

*• Creatinine kinase elevation in alcohol intoxicated patients may be as a result of direct “muscular” toxicity” (myotoxicity) or from prolonged immobilization and ischemic compression induced by coma.*

**What is New:**

*• Our retrospective cohort study is a pioneer in addressing the effect of acute alcohol intoxication amongst adolescents (< 18 years) upon muscle tissue (creatinine kinase level) within a large population. When evaluating the blood samples of the included population, elevated creatinine kinase levels were found in 60% of the cases, with a mean of 254 U/I (normal value ≤ 145 U/I).*

*• There is an association between alcohol intoxication and elevated creatinine kinase levels amongst adolescents. Future research is needed to further understand the pathophysiology and causality of this interaction.*

## Introduction

The World Health Organization stated that most adolescents between the age of 15 and 24 drink alcohol in heavy drinking sessions [1]. These sessions, which are also referred to as binge drinking, can lead to acute alcohol intoxication (AAI), which is an ongoing concern amongst adolescents [2]. Alcohol use specifically in adolescence is associated with adverse psychological, social, and physical health consequences [3, 4].

In the Netherlands, it is illegal to buy and/or consume alcoholic beverages < 18 years old. According to literature, sports canteens/bars are an outlets of major concern because the ID control compliance rates are low [5]. Therefore, it will be interesting to look at muscle related consequences of AAI to potentially help prevent AAI within the sports world. Amongst others, rhabdomyolysis has been identified as a complication of AAI in extant literature [6–9]. However, there is no literature examining the prevalence of rhabdomyolysis amongst adolescents after AAI.

Rhabdomyolysis, acute muscle fibre necrosis, is accompanied by the leakage of muscle constituents into the blood [10]. The diagnosis is confirmed as an increase in creatine kinase (CK) to the degree of 5 − tenfold the upper limit of normal [11]. Alcohol, alongside pharmaceutical agents and illicit drugs, is a significant cause of rhabdomyolysis. In AAI, CK elevation may be as a result of direct “muscular” toxicity” (myotoxicity) or from prolonged immobilization and ischemic compression induced by coma [8, 9]. The outcomes following rhabdomyolysis vary, ranging from asymptomatic elevations of CK concentration to life-threatening electrolyte abnormalities and acute kidney injury (AKI) [12].

The Reinier de Graaf Gasthuis (RdGG), a major district general hospital in the Netherlands, took a pioneering role in the prevention of AAI amongst Dutch adolescents when it opened a primary outpatient clinic for ‘adolescents and alcohol’ in 2006. When patients < 18 years of age were admitted at the emergency department of the RdGG upon suspicion of AAI, blood tests were carried out, including their CK level. The primary objective of this study is to determine the prevalence of CK-elevation amongst a sample of Dutch adolescents admitted for AAI.

## Methods

### Participants

In the RdGG, a major district general hospital in Delft, the Netherlands, underage (< 18 years old) patients with AAI (positive blood alcohol concentration (BAC) and/or clinical features of alcohol intoxication) during the period 2008 to 2021 were included in the study. This included patients who gave consent for data collection using the Paediatric alcohol questionnaire [13], while additional relevant non-identifiable information about the AAI event was retrospectively collected via the diagnostic treatment code ‘alcohol intoxication’.

## Materials

The primary objective of this study was to determine the prevalence of rhabdomyolysis amongst adolescents with AAI in the RdGG. Therefore, the CK level was evaluated for each patient with AAI.

The secondary objectives were to determine whether characteristics such as gender, mean age, mean BAC, mean Glasgow coma score (EMV) score and/or proportion of positive drug screenings differed across those patients with elevated and normal CK levels. Sociodemographic characteristics such as age and gender were the continuous variables, and therefore a mean with standard deviation (SD) was given. The intoxication characteristics such as BAC and positive drug screenings were expressed in gram/liter (g/l) and in % of the total population, respectively.

Moreover, the standard blood tests carried out on patients admitted with AAI includes the following: sodium (in mmol/l), potassium (in mmol/l), calcium (in mmol/l), glucose (in mmol/l), chloride (in mmol/l), CK (in U/l), urea (in mmol/l), creatinine (in µmol/l), aspartate aminotransferase (ASAT, in U/l), alanine aminotransferase (ALAT, in U/l), gamma-glutamyl transferase (GGT, in U/l), bilirubin (in µmol/l), alpha-fetoprotein (in U/l), lactate dehydrogenase (LDH, in U/l), blood pH, bicarbonate (HCO3^−^, in mmol/l), and pCO2 (in kPa). The central laboratory for analysis of these samples is the Reinier Medical Diagnostic Centre. During the study period, the Cobas analyser (Roshe firm) was used for laboratory tests during the entire sturdy period.

These blood values are shown in Table 1, along with the normal value range, the mean and the proportion (in %) of reduced or elevated values within the population.

**Table 1:** Population characteristics with blood sample

		**Girls**	**Boys**	**Total population**
Amount of adolescents		277 (56%)	218 (44%)	495
Median age, IQR (years, *n* = 495)		15 (1)	16 (1)	16 (1)
Mean BAC, sd (g/l, *n* = 493)		0.19 (0.06)	0.20 (0.05)	0.19 (0.06)
Median EMV-score, IQR (*n* = 181)		14 (3)	15 (3)	15 (3)
Proportion of substance abuse, CI (*n*, %, *n =* 444)		11 (4.6%)	29 (15.4%)	40 (9%)
**Blood sample with normal value in SI-unit**	***N***	**Mean (SD)**	**Below lower limit of normal (*n*, %)**	**Above higher limit of normal (*n*, %)**
Sodium (135 − 145 mmo/l)	486	142 (3)	2 (0.4%)	39 (8%)
Potassium (3.2 − 4.7 mmol/l)	478	3.8 (0.4)	24 (5%)	16 (3.2%)
Calcium (2.20 − 2.65 mmol/l)	464	2.3 (0.1)	90 (19%)	0 (0%)
Chloride (97 − 107 mmol/l)	469	106 (3)	2 (0.4%)	178 (38%)
CK (≤ 145 U/I)	458	254 (321)	–	275 (60%)
Urea (1.8 − 6.4 mmol/l)	478	3.9 (1.1)	5 (1.0%)	4 (0.8%)
Creatinine (40 − 68 µmol/l)	477	65 (11)	5 (1.0%)	171 (36%)
ASAT (0 − 31 U/L)	450	26 (9)	–	71 (16%)
ALAT (< 34 U/L)	470	23 (9)	–	43 (9.1%)
GGT (< 38 U/L)	477	15 (7)	–	7 (1.5%)
Bilirubin (< 21 µmol/l)	465	7 (5)	–	12 (2.6%)
Alkaline phosphatase (0 − 500 U/L)	464	123 (73)	–	0 (0%)
LDH (< 247 U/l)	419	199 (36)	–	34 (8.1%)
pH (7.35 − 7.45)	364	7.36 (0.58)	149 (41%)	27 (7.4%)
Bicarbonate (HCO_3_-, 21 − 28 mmol/l)	466	22 (3)	134 (29%)	4 (0.9%)
pCO_2_ (4.3 − 6.0 kPa)	325	5.3 (1.0)	38 (12%)	48 (15%)

To assess the effects of BAC on CK levels, we used logistic regression models. First, we ran the analyses crude. Next, we ran the analyses adjusted for relevant covariates. Here, we selected covariates that had a 10% effect on the beta coefficients of the original analyses. We report *p* values, beta coefficients, and 95% confidence intervals (CI).

### Procedures

All anonymous data were transformed in an SPSS dataset (version 25) for the purposes of the analyses. Descriptive statistics were used to show the baseline characteristics of the study population. Proportions were expressed as percentages, with 95% CIs. All continuous data were expressed as the average with SD. Those CIs that did not include one were considered to be statistically significant. The significance level was set to *p* = 0.05.

Both the medical ethical commission Leiden-Den Haag-Delft (*METC code G1.192*) and the research committee of the RdGG approved the manner of data collection adopted in this study.

## Results

Overall, 506 adolescents < 18 years were admitted to the emergency department of the RdGG during the period 2008 − 2021 with the diagnostic treatment code ‘alcohol intoxication’. In seven cases, there was a misclassification of the diagnostic treatment code, and no alcohol intoxication was reported in the electronic patient file, and therefore these patients were excluded from the study. Moreover, blood tests had not been performed on four patients, due to, amongst other things, resistance from the patient, and thus these patients were also excluded. Overall, 495 patients were included in this study. Five patients were admitted on two separate occasions to the emergency department with AAI, and so both events were included, because a new AAI event had taken place.

Of the 495 patients, 277 (56%) were girls (see Table 1). The median age of the entire study population was 16 years old, with girls being significantly younger than boys at the time of admission, aged 15 and 16, respectively (*p* = 0.003). The youngest children in the study were 12 years old (*n* = 4). The mean BAC was 0.19 g/l.

When evaluating the blood samples of the included patients, elevated CK levels were found in 60% (*n* = 275) of cases, with a mean of 254 U/I (see Table 1). The confirmed diagnosis rhabdomyolysis (increase in CK > fivefold the upper limit of normal) was present in 4.4% of cases (*n* = 21), with the highest value being 3458 U/I. The creatinine was elevated in 36% (*n* = 171) of the included adolescents with AAI. Moreover, the blood pH was lower than 7.35 in 41% of cases. Within the acidotic patients, 28% had a bicarbonate < 21 mmol/l and 31% had a pCO2 > 6.0 kPa. The chloride levels were elevated in 38% of the patients. The liver panel was in the normal range within this population (ASAT mean 26 U/I, ALAT mean 23 U/I, LDH 198 U/L).

When using a linear regression model to analyse the effects of a higher BAC on CK levels, we did not find a statistically significant association (*p* = 0.089; *β* = 49.42; 95% CI −7,483 –104.31). However, when adjusted for positive drug screening, we found that a higher BAC was associated with higher CK levels (*p* = 0.027; *β* = 66.88; 95% CI 7.68 − 126.08).

## Discussion

Of the 495 adolescents with AAI included in our study, 60% (*n* = 275/458) showed elevated CK levels in their blood samples. Rhabdomyolysis (increase in CK > fivefold the upper limit of normal) was present in 4.4% of cases (*n* = 21/458). Consequently, most of the adolescents with AAI were in the preliminary stage of rhabdomyolyses with elevated CK levels (< fivefold the upper limit of normal). It is important to know that CK elevation is present within patients in order to prevent extensive rhabdomyolysis from occurring [8]. Therefore, when patients are admitted to an emergency department with AAI, they should always undergo blood and urine tests for early recognition and treatment of rhabdomyolysis [14]. The treatment aims at the discontinuation of further skeletal muscle damage, the prevention of acute renal failure, and rapid identification of potentially life-threatening complications, such as hyperkalaemia and compartment syndrome [11]. Intravenous fluids should be initiated as soon as possible, preferably within the first 6 hours after experiencing muscle injury. Furthermore, using a linear regression this study found that a higher BAC was associated with higher CK levels, when adjusted for positive drug screenings amongst adolescents with AAI (*p* = 0.027; *β* = 66.88; 95% CI 7.68 − 126.08). The combination of elevated CK levels and the fact that the liver panel is mostly in the normal range within this population could be a sign of muscle breakdown.

There can be multiple contributing factors leading to CK elevation amongst patients with AAI than merely alcohol intoxication itself, including, amongst other things, trauma [11], heavy sports training prior to drinking [15], or immobilisation (coma) during hospital admission for AAI. The risk of accidents that lead to trauma increases when alcohol has been consumed, primarily due to reduced coordination, reaction times, and concentration. A recent study based on the entire Dutch population of AAI < 18 years old from 2007 to 2017 addressed the reason for admittance to emergency departments [16]. The category ‘accident, fracture or suicide attempt’ was given as the reason for admission in 11% of cases. Consequently, we presume that the CK elevation observed amongst 60% of our population cannot only be explained by trauma (which was present in 11% of a comparable population [16]). Therefore, we presume that the alcohol intoxication itself must have contributed to elevated CK levels amongst this population. Conversely, heavy sports training prior to the emergency department visit can also result in elevated CK levels. Indeed, extant literature shows that CK elevation can still be visible 72 hours post-workout [17]. However, the cut-off point for the serum creatinine kinase upper limit in this literature often differs from our study. For instance, rather than > fivefold, as used in our study, one study used two-times the upper limit of normal for CK [18], which might have resulted in more extensive outcomes. Although we do not have data regarding the patients’ sporting activities prior to their admission to the emergency department, we do know that 75% of adolescents aged between 12 and 17 engage in sport more than one time per week [19]. The immobilisation factor amongst adolescents with AAI can also influence CK elevation. However, the median EMV score amongst our population (reported in *n* = 181) was 15. This indicates that at the time the blood tests were carried out, it was unlikely that there were any signs of a coma that may result in immobilisation.

There were other interesting blood results. As expected, creatinine, which point out the degree of kidney damage (either pigment induced or secondary to fluid volume depletion), was elevated in 36% (*n* = 171) of the included adolescents with AAI. Moreover, the blood pH < 7.35 was present in 41% of cases. Acidosis may result either from hypoventilation (respirator) or from metabolic causes in the adolescents with AAI. In our population of 149 patients with an acidosis, the cause was respirator in 31% (*n* = 46 with pCO2 > 6.0 kPa) and metabolic in 28% (*n* = 41 with bicarbonate < 21 mmol/l).

The chloride levels were elevated in 38% of our population. The aforementioned article focusing on AAI amongst Dutch adolescents in 2000 − 2010 also found hyperchloremia in 31% of their patients [20]. However, they also found low bicarbonate (22%), hypokalemia (12%), and hypernatremia (8%). The hypernatremia was comparable and the hypokalemia was not as prominent in our study, while bicarbonate was < 21 mmol/l in 29% of our patients (*n* = 134).

Our retrospective cohort study is a pioneer in addressing the effect of AAI amongst adolescents upon muscle tissue (CK level) within a large population. Prior to this study, there have been only a couple of case reports published on this topic. Based on our study, we can conclude that there is a clear association between alcohol intoxication and elevated CK levels amongst adolescents < 18 years old. Future research is needed to further understand the pathophysiology and causality of this interaction. Due to the lack of ID control in Dutch sports canteens, there are less barriers to underage alcohol consumption [5]. Therefore, we hope that our results can potentially help prevent alcohol intoxication within the sports world.

## Data Availability

The data that support the findings of this study are not openly available.

## Abbreviations

AAI: Acute alcohol intoxication
AKI: Acute kidney injury
BAC: Blood alcohol concentration
CI: Confidence interval
CK: Creatine kinase
EMV: Glasgow coma score
RdGG: Reinier de Graaf Gasthuis
sd: Standard deviation
